# Bayesian analysis for social data: A step-by-step protocol and interpretation

**DOI:** 10.1016/j.mex.2020.100924

**Published:** 2020-05-19

**Authors:** Quan-Hoang Vuong, Viet-Phuong La, Minh-Hoang Nguyen, Manh-Toan Ho, Trung Tran, Manh-Tung Ho

**Affiliations:** a Centre for Interdisciplinary Social Research, Phenikaa University, Yen Nghia Ward, Ha Dong District, Hanoi 100803, Vietnam; b A.I. for Social Data Lab, Vuong & Associates, 3/161 Thinh Quang, Dong Da District, Hanoi, 100000, Viet Nam; c Vietnam Academy for Ethnic Minorities, Hanoi 100000, Vietnam

**Keywords:** Bayesian statistics, Social data, Markov chain monte carlo (MCMC), Bayesvl

## Abstract

The paper proposes Bayesian analysis as an alternative approach for the conventional frequentist approach in analyzing social data. A step-by-step protocol of how to implement Bayesian multilevel model analysis with social data and how to interpret the result is presented. The article used a dataset regarding religious teachings and behaviors of lying and violence as an example. An analysis is performed using R statistical software and a bayesvl R package, which offers a network-structured model construction and visualization power to diagnose and estimate results.

• *The paper provides guidance for conducting a Bayesian multilevel analysis in social sciences through constructing directed acyclic graphs (DAGs, or "relationship trees") for different models, basic and more complex ones.*
• *The method also illustrates how to visualize Bayesian diagnoses and simulated posterior.*
• *The interpretations of visualized diagnoses and simulated posteriors of Bayesian inference are also discussed.*

## Graphical abstract

**Figure:**
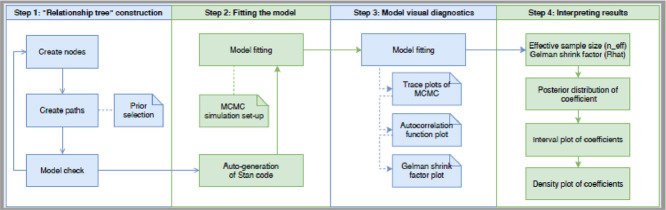

Specifications table

**Table:** 

Subject Area	Psychology
More specific subject area	
Method name	*Bayesian statistics*
Name and reference of the original method	*Hamiltonian MCMC*
Resource availability	*R statistical software: https://www.r-project.org/*
	*Bayesvl R package: https://cran.r-project.org/web/packages/bayesvl/index.html*
	Data: https://github.com/sshpa/bayesvl/tree/master/data

## Method details

In social sciences, the persistence of 'stargazing', p-hacking, and HARKing issues has currently led to a severe reproducibility crisis in which 70% of researchers have failed to reproduce the experiments of other scientists [1], [2], [3], [4]. The crisis forces the academia to react with rigorous study design and preregistration procedures, more careful use of statistical analysis, and interpretation of statistical results [5], [6], [7]. In this article, we propose that the Bayesian inference approach [8,9], with its natural properties, seemingly offers a solution for analyzing social data. In the following section, we will briefly explain a dataset of Vietnamese folktales that we are going to use as an example to illustrate the method.

The analysis was done using the bayesvl R package (version 0.8.5) in the R statistical software (version 3.6.2) [10]. Similar applications of Bayesian statistics in social data analysis can be found in [11], [12], [13], [14].

## Data in brief

Hereafter, we use one of our latest research studies as an example for performing Bayesian multilevel analysis with social data [14]. The study explores the association between the outcome and the behaviors of lying and violence of main characters under the influence of religious teachings in selected Vietnamese folktales. The dataset consists of binary variables encoded from 307 Vietnamese folktales. The dataset is stored in the bayesvl repository and can be loaded with the following commands:

*R*> data(Legends345)
*R*> data1 <- Legends345
*R*> head(data1)

Even though there are 25 binary variables, of which only eight variables are employed in this article:

• "Lie": whether the main character lies
• "Viol": whether the main character employs violence
• "VB": whether the main characters' behaviors express the value of Buddhism
• "VC": whether the main characters' behaviors reflect the value of Confucianism
• "VT": whether the main characters' behaviors express the value of Taoism
• "Int1": whether there are interventions from the supernatural world
• "Int2": whether there are interventions from the human world
• "Out": whether the outcome of a story is favorable for its main characters

## Data analysis with Bayesian statistics

### Step 1. model construction

First, we establish three different directed acyclic graphs (DAGs), or so-called "relationship trees," from simple to more complex ones, based on the dataset mentioned above.

#### Model 1. Multiple regression analysis

**Fig. 1:**
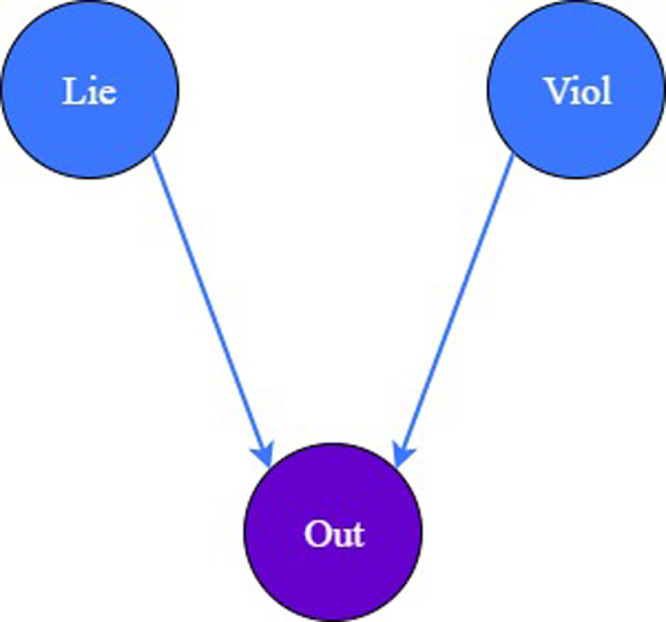
The "relationship tree" of model 1.

The first and the most straightforward "relationship tree" exemplified examines the determinants of the behaviors of lying and violence on the outcome of the main character (see Fig. 1).

To construct the "relationship tree" in Fig. 1, one needs to initially create the model and load the variables – represented by nodes – into the model by employing the function bayesvl() and bvl_addNode(), respectively as follows:

*R*> library(bayesvl)
*R*> model1 <- bayesvl()
*R*> model1 <- bvl_addNode(model1, "O", "binom")
*R*> model1 <- bvl_addNode(model1, "Lie", "binom")
*R*> model1 <- bvl_addNode(model1, "Viol", "binom")

Because the statistical distribution of all employed variables is binomial, we set "binom" in the function. Besides binomial distribution, the package also provides various types of statistical distribution for the types of data, namely: normal distribution – "norm", categorical distribution – "cat", Bernoulli distribution – "bern", Student's t-distribution – "student", Poisson distribution – "pois", and so on.

After loading all the variables into the "relationship tree", the next step is to grant the regression type to the connection between the independent variables "Lie" and "Viol" and the dependent variable "O" by employing the function bvl_addArc(). The model can be set as the fixed effect type by adding a "slope" into the command:

*R*> model1 <- bvl_addArc(model1, "Viol", "O", "slope")
*R*> model1 <- bvl_addArc(model1, "Lie", "O", "slope")

In Bayesian inference, the posterior probability is estimated from a prior probability and a "likelihood function" derived from a statistical model for the observed data. Therefore, setting prior distribution is critical before fitting the model. The prior distribution can be determined based on previous empirical findings, researcher's past experience and personal intuition, or expert opinion [8,15]. Nonetheless, preceding empirical works and knowledge do not always exist, so determining prior distribution by researcher's experience or personal intuition might be criticized as intentional subjectivity. In such circumstances, setting prior distribution as “uninformative” or “know nothing priors” can be a prominent alternative, because it can mitigate the criticism of intentional subjectivity and help users fit a new model without firm empirical findings [15]. The package developers utilize uninformative prior distribution with mean 0 and standard deviation 10 (or 100 for alpha) as default. The prior distribution of each relationship in the "relationship tree" is always given at the time the path between two nodes is created employing the function bvl_addArc(), but if the prior distribution is not set, the package will use the default prior distribution. The prior distribution setting method will be clearly explained when constructing model 3 below. One can check the prior distribution of coefficients in model 1 by typing:

*R*> bvl_stanPriors(model1)
a_O ~ normal(0,100)
b_Viol_O ~ normal(0, 10)
b_Lie_O ~ normal(0, 10)

**Fig. 2:**
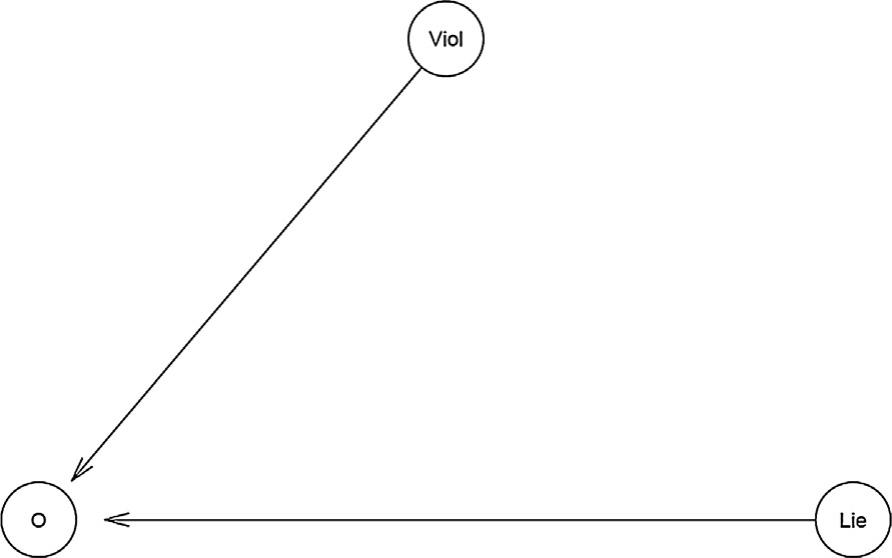
The "relationship tree" of model 1 generated by the package.

Since the prior distribution was not set in bvl_addArc(model1, "Viol", "O", "slope"), the package automatically set prior distribution of b_Viol_O as default distribution which is normal(0, 10). Eventually, the function bvl_bnPlot can help produce the graphical network of the constructed model (see Fig. 2).

*R*> bvl_bnPlot(model1)

To check the structure and mathematical form of the model, one can use the function summary:

*R*> summary(model1)
Model Info:
nodes: 3
arcs: 2
scores: NA
formula: O ~ a_*O* + *b*_Lie_O * Lie + *b*_Viol_O * Viol
Estimates:
model is not estimated.

#### Model 2. multiple regression analysis with interaction variables

**Fig. 3:**
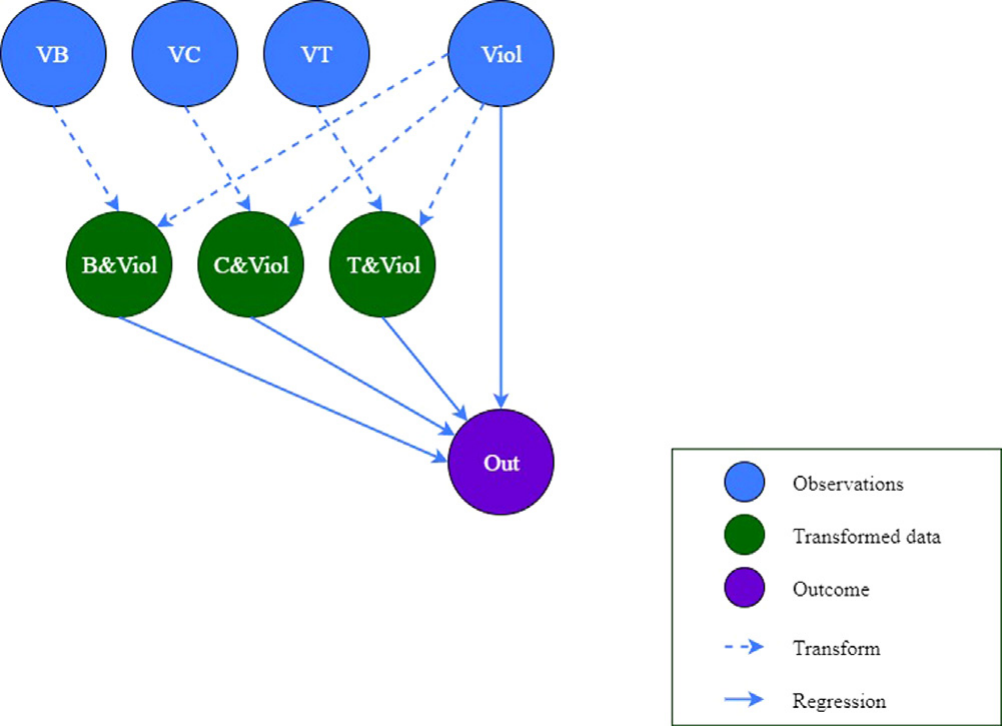
The "relationship tree" of model 2.

The second "relationship tree" is designed to estimate the impact of violent behavior and its interaction effect with religious values on the outcome of the main character (see Fig. 3). Similar to the first "relationship tree", a model and variables are created and inserted into the model using two functions bayesvl() and bvl_addNode(), respectively:

*R*> model2 <- bayesvl()
*R*> model2 <- bvl_addNode(model2, "O", "binom")
*R*> model2 <- bvl_addNode(model2, "Viol", "binom")
*R*> model2 <- bvl_addNode(model2, "VB", "binom")
*R*> model2 <- bvl_addNode(model2, "VC", "binom")
*R*> model2 <- bvl_addNode(model2, "VT", "binom")
*R*> model2 <- bvl_addNode(model2, "B_and_Viol", "trans")
*R*> model2 <- bvl_addNode(model2, "C_and_Viol", "trans")
*R*> model2 <- bvl_addNode(model2, "T_and_Viol", "trans")

The variables "B_and_Viol", "C_and_Viol", and "T_and_Viol" are the interaction variables between the act of violence and the value of Buddhism, Confucianism, and Taoism, respectively. The independent interaction variables, represented by the green nodes, can be subsequently created from two normal independent variables, represented by the blue nodes. Unlike the normal variable "Viol" defined as "binom", or binomial, the interaction variables are defined as "trans", or interaction/transformed. It is noteworthy that the "trans" variable does not have a particular distribution but depends on the interaction of two normal variables through applying " * " or " + " operator. To standardize, we call normal independent variables as observation data and interaction variables as transformed data from now on.

The dash-line arrow demonstrates the relation between the transformed data and the observation data (see Fig. 3). The values of transformed data are generated from the values of two observation data through the mathematical operator " * ". The value of "B_and_Viol" is generated from the multiplication between the values of "VB" and "Viol" by using the function bvl_addArc(). One can use a similar function to give the transformed value to "C_and_Viol" and "T_and_Viol".

*R*> model2 <- bvl_addArc(model2, "VB", "B_and_Viol", "*")
*R*> model2 <- bvl_addArc(model2, "Viol", "B_and_Viol","*")

The model can be set as the fixed effect type by adding "slope" into the command:

*R*> model2 <- bvl_addArc(model2, "Viol", "O", "slope")
*R*> model2 <- bvl_addArc(model2, "B_and_Viol", "O", "slope")
*R*> model2 <- bvl_addArc(model2, "C_and_Viol", "O", "slope")
*R*> model2 <- bvl_addArc(model2, "T_and_Viol", "O", "slope")

The prior distributions of model 2 are also set as default:

a_O ~ normal(0,100)
b_Viol_O ~ normal(0, 10)
b_B_and_Viol_O ~ normal(0, 10)
b_C_and_Viol_O ~ normal(0, 10)
b_T_and_Viol_O ~ normal(0, 10)

**Fig. 4:**
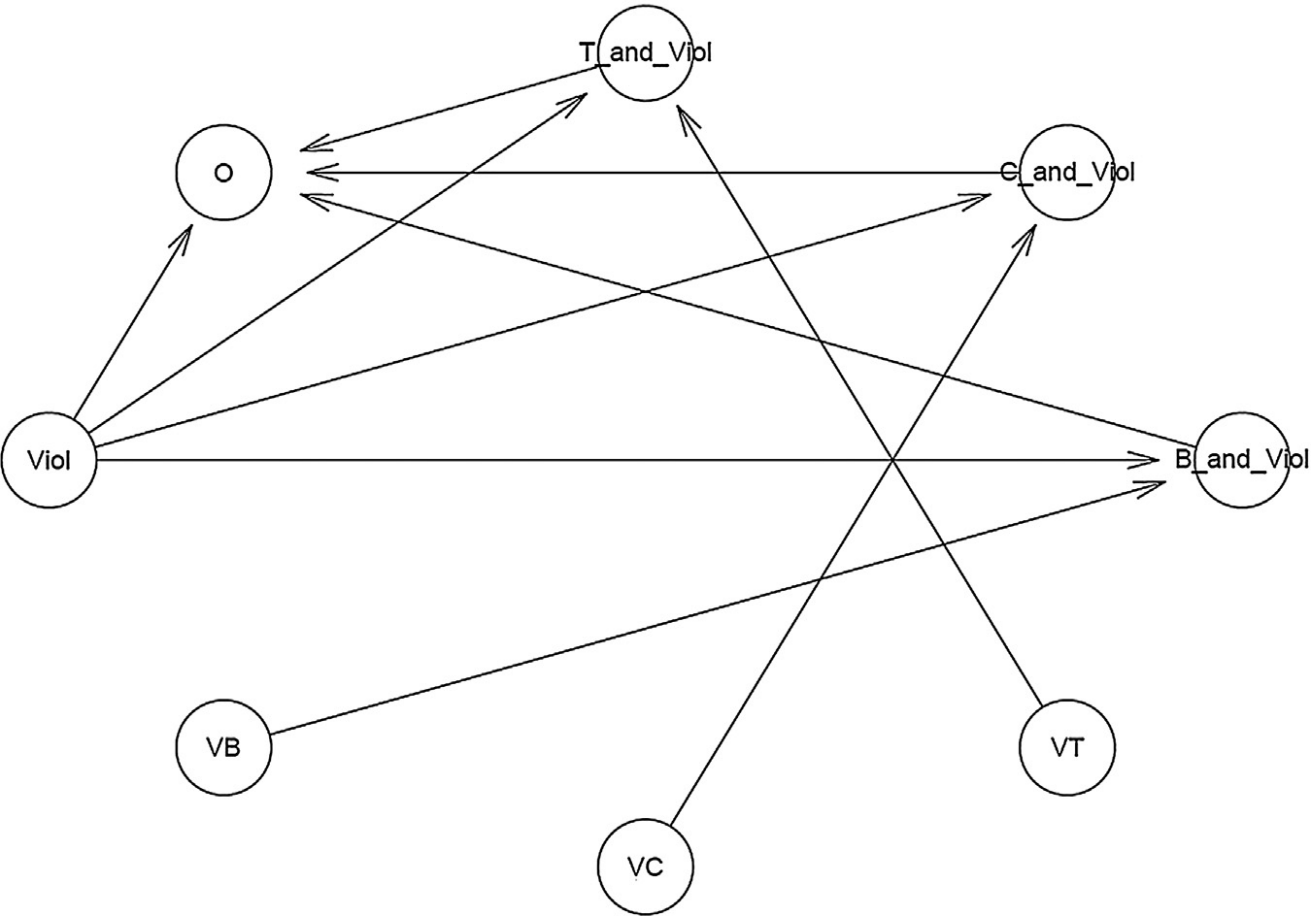
The "relationship tree" of model 2 generated by the package.

Eventually, the function bvl_bnPlot() and summary() can help produce the graphical network (see Fig. 4) and the mathematical form of the constructed model, respectively.

*R*> bvl_bnPlot(model2)
*R*> summary(model2)
Model Info:
nodes: 8
arcs: 9
scores: NA
formula: O ~ a_*O* + *b*_B_and_Viol_O * VB*Viol + *b*_C_and_Viol_O * VC*Viol + *b*_T_and_Viol_O * Viol*VT
Estimates:
model is not estimated.

#### Model 3. multilevel regression analysis

**Fig. 5:**
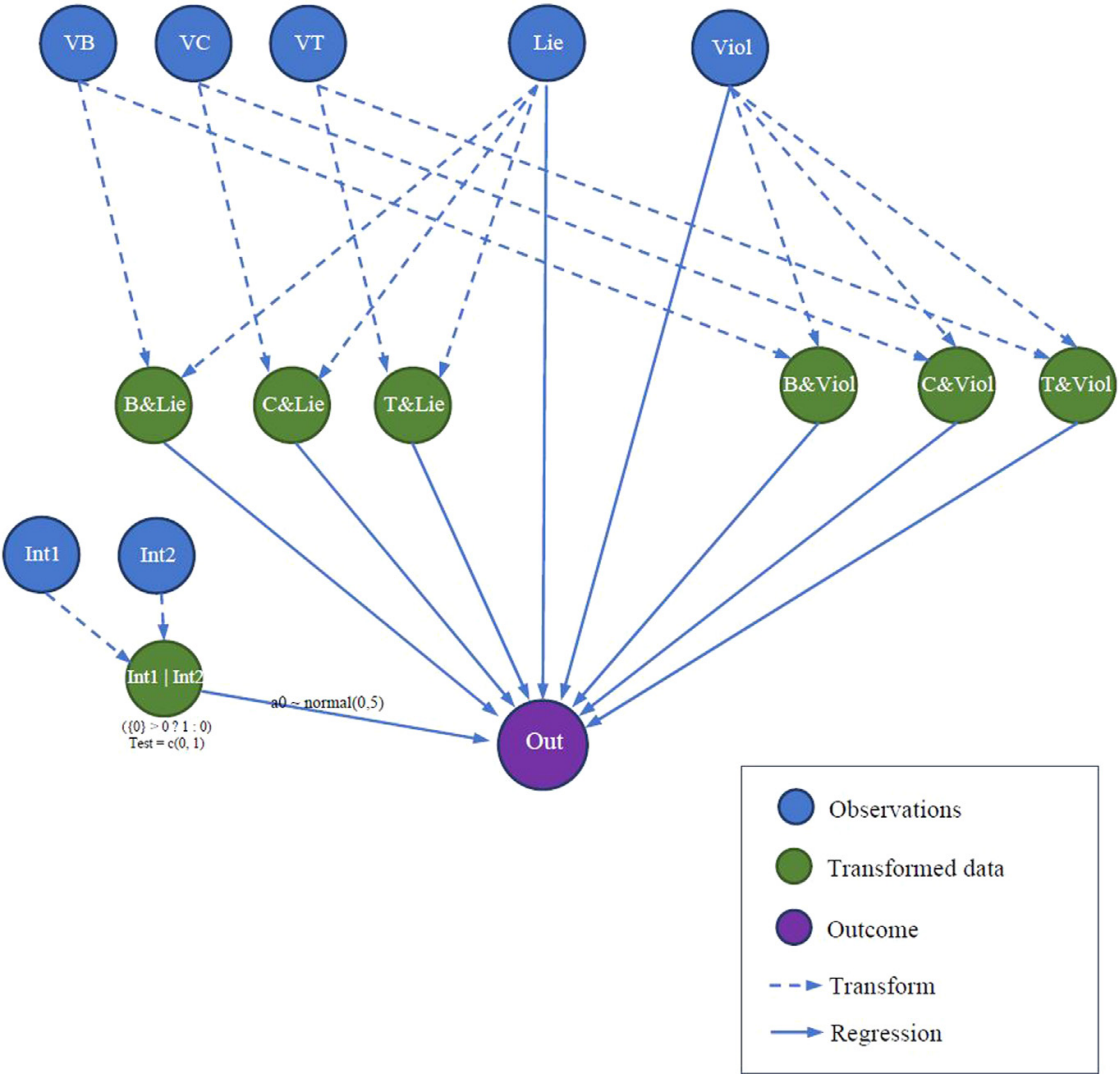
The "relationship tree" of model 3.

One can create a much more complex model of multilevel regression analysis, while only following a similar procedure with two models mentioned above and employing some additional functions. The primary purpose of the third exemplary "relationship tree" is to explore the impacts of lying and violence behaviors, their interaction with religious values, and intervention from the supernatural or human world on the outcome of the main character (see Fig. 5).

To construct the "relationship tree" illustrated in Fig. 5, the functions bayesvl(), bvl_addNode(), and bvl_addArc() are used comparably similar to model 1 and model 2 above. Notably, to conduct the multilevel regression analysis between the outcome "O" and the transformed data "Int1_or_Int2", things become a little more complicated. The transformed data "Int1_or_Int2" is generated from observational data "Int1" and "Int2" applying the following conditional algorithm:

Int1_or_Int2 = (Int1 + Int2 > 0 ? 1: 0)

Therefore, the command to create the node of "Int1_or_Int2" is augmented as follows:

R> model3 <- bvl_addNode(model3, "Int1_or_Int2", "trans",
+ fun = "({0}> 0 ? 1: 0)", out_type = "int", lower = 0, test = c(0, 1))

fun = "({0}> 0 ? 1: 0)" is equivalent to the conditional algorithm shown above, while out_type stands for the property of the output, such as "int" (integer) and "real" (real number). The parameter test = c(0, 1) helps to insert the code computing “fixed predicted outcome” when Int1_or_Int2 = 0 and Int1_or_Int2 = 1. The value of transformed data "Int1_or_Int2" is defined based on the values of observational data "Int1" and "Int2" through the mathematical operator " + ":

*R*> model3 <- bvl_addArc(model3, "Int1", "Int1_or_Int2", "+")
*R*> model3 <- bvl_addArc(model3, "Int2", "Int1_or_Int2", "+")

For completing the "relationship tree" construction, the last step is to connect two observational data "Lie" and "Viol" as well as other transformed data to the outcome "O". Like previous commands, the function bvl_addArc() is used, but "trans" is replaced by "slope" (fixed effect) or "varint" (varying intercept), to convert the relationships between "O" and other nodes into regression relationships. There are four fundamental types of statistical model integrated in the bayesvl package: fixed-effect model ("slope"), varying-intercept model ("varint"), varying-slope model ("varslope"), and mixed-effect model ("varpars").

*R*> model3 <- bvl_addArc(model3, "Viol", "O", "slope")
*R*> model3 <- bvl_addArc(model3, "B_and_Viol", "O", "slope")
*R*> model3 <- bvl_addArc(model3, "Int1_or_Int2", "O", "varint",
+ priors = *c*("a0_ ~ normal(0,5)", "sigma_ ~ normal(0,5)"))

The first and second commands are to create the regression relationships of the outcome with observational and transformed data, respectively, employing a fixed-effect model, while the third command is to create the regression relationship between the outcome and transformed data employing a varying-intercept model. In model 3, the prior distribution of all the paths from observational and transformed nodes to the outcome node is set as default, except for the path from "Int1_or_Int2" to "O". The prior distributions of the relationship between "Int1_or_Int2" and "O" is set by adding the code priors = *c*("a0_ ~ normal(0,5)", "sigma_ ~ normal(0,5)") into the function bvl_addArc(). Similarly, this method can be applied to change the prior distribution of other relationships by using the prefix a0_, b_, or sigma_, depending on the relationship type. Besides normal distribution, other kinds of distribution can also be implemented for setting up prior distribution by replacing "normal" by the name of the designated distribution (e.g. binomial and beta, etc.). The prior distribution of each path can be checked by typing:

*R*> bvl_stanPriors(model3)
b_B_and_Viol_O ~ normal(0, 10)
b_C_and_Viol_O ~ normal(0, 10)
b_T_and_Viol_O ~ normal(0, 10)
b_Viol_O ~ normal(0, 10)
b_B_and_Lie_O ~ normal(0, 10)
b_C_and_Lie_O ~ normal(0, 10)
b_T_and_Lie_O ~ normal(0, 10)
b_Lie_O ~ normal(0, 10)
a0_Int1_or_Int2 ~ normal(0,5)
sigma_Int1_or_Int2 ~ normal(0,5)
u_Int1_or_Int2 ~ normal(0, sigma_Int1_or_Int2)

**Fig. 6:**
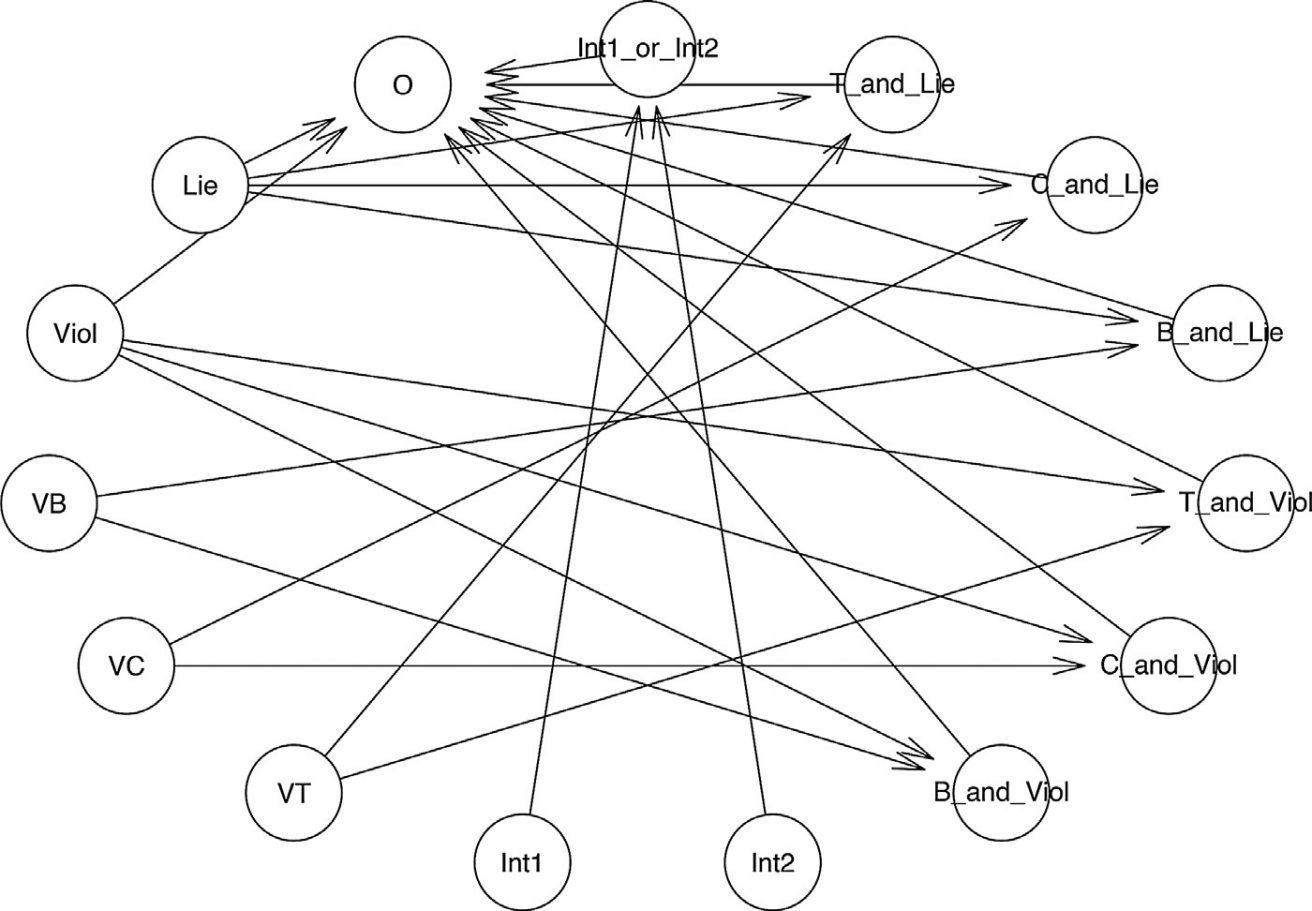
The "relationship tree" of model 3 generated by the package.

Eventually, the function bvl_bnPlot() can help produce the graphical network of the constructed model (see Fig. 6).

*R*> bvl_bnPlot(model3)

One can also check the mathematical construct of each transformed data in the "relationship tree" above by using the function bvl_formula(), like the following examples:

*R*> bvl_formula(model3, "B_and_Lie")
B_and_Lie ~ VB*Lie
*R*> bvl_formula(model3, "Int1_or_Int2")
Int1_or_Int2 ~ (Int1+Int2 > 0 ? 1: 0)

To check the structure and mathematical form of the model, one can use the function summary():

*R*> summary(model3)
Model Info:
nodes: 15
arcs: 23
scores: NA
formula: O ~ b_B_and_Viol_O * VB*Viol + *b*_C_and_Viol_O * VC*Viol + *b*_T_and_Viol_O * VT*Viol + *b*_Viol_O * Viol + *b*_B_and_Lie_O * VB*Lie + *b*_C_and_Lie_O * VC*Lie + *b*_T_and_Lie_O * VT*Lie + *b*_Lie_O * Lie + *a*_Int1_or_Int2[(I*nt*1+I*nt*2 > 0 ? 1: 0)]

Estimates: model is not estimated!

### Step 2. Fitting the model

Before fitting the model using MCMC simulation, one needs to generate the Stan code in R. Because the bayesvl package provides an automatic generation of Stan code, one can use the following commands:

*R*> model_string <- bvl_model2Stan(model3)
*R*> cat(model_string)

The model created from the "relationship tree" can be fitted with MCMC simulation using the function bvl_modelFit(). The structure of the function bvl_modelFit() is partly dissimilar with other currently existent Bayesian analysis packages because it does not require users to construct conventional mathematical relationships among variables as well as set up the prior distribution for each relationship. One only need to input the name of constructed "relationship tree", the dataset, and mandatory set-up for MCMC simulation. As the bayesvl package was coded utilizing the No-U-Turn Sampler (NUTS) sampler [16], the effective sample size per iteration is usually higher than that utilizing other samplers. However, the simulation is more computationally intensive and time-consuming. Thus, it should be aware that the model specified with a high number of iterations, chains, and cores might monopolize computing power for a substantial time, especially for less powerful machines The command for model fit in the current exemplary case is shown below:

*R*> model3 <- bvl_modelFit(model3, data1, warmup = 2000, iter = 5000, chains = 4, cores = 4)
*R*> summary(model3)
Model Info:
nodes: 15
arcs: 23
scores: NA
formula: O ~ b_B_and_Viol_O * VB*Viol + *b*_C_and_Viol_O * VC*Viol + *b*_T_and_Viol_O *VT*Viol
+ *b*_Viol_O * Viol + *b*_B_and_Lie_O * VB*Lie + *b*_C_and_Lie_O * VC*Lie + *b*_T_and_Lie_O * VT*Lie
+ *b*_Lie_O * Lie + *a*_Int1_or_Int2[(I*nt*1+I*nt*2 > 0 ? 1: 0)]
Estimates:
Inference for Stan model: d4bbc50738c6da1b2c8e7cfedb604d80.
4 chains, each with iter=5000; warmup=2000; thin=1;
post-warmup draws per chain=3000, total post-warmup draws=12,000.

**Table:** 

	mean	se_mean	sd	2.5%	25%	50%	75%	97.5%	n_eff	Rhat
b_B_and_Viol_O	2.55	0.05	1.46	0.13	1.50	2.41	3.42	5.73	915	1.01
b_C_and_Viol_O	–0.28	0.01	0.61	–1.46	–0.68	–0.31	0.13	0.93	6689	1.00
b_T_and_Viol_O	–0.96	0.01	1.09	–3.21	–1.65	–0.91	–0.26	1.14	6820	1.00
b_Viol_O	–0.62	0.01	0.42	–1.43	–0.90	–0.62	–0.35	0.23	5892	1.00
b_B_and_Lie_O	0.70	0.02	1.44	–1.78	–0.28	0.56	1.52	4.03	6546	1.00
b_C_and_Lie_O	1.47	0.02	0.68	0.21	0.97	1.45	1.94	2.86	1676	1.01
b_T_and_Lie_O	2.23	0.02	1.59	–0.41	1.10	2.06	3.16	5.85	4523	1.00
b_Lie_O	–1.05	0.01	0.37	–1.77	–1.30	–1.05	–0.81	–0.32	3984	1.00
a_Int1_or_Int2[1]	1.20	0.00	0.21	0.78	1.05	1.20	1.33	1.62	7767	1.00
a_Int1_or_Int2[2]	1.35	0.00	0.19	0.99	1.23	1.35	1.48	1.73	3512	1.00
a0_Int1_or_Int2	1.18	0.04	1.34	–1.91	0.87	1.25	1.57	3.83	1353	1.00
sigma_Int1_or_Int2	1.49	0.04	1.82	0.04	0.28	0.78	1.98	6.67	1759	1.00

The model is fitted using four chains, each with 5000 iterations of which the first 2000 are for warmup, resulting in a total of 12,000 post-warmup posterior samples. In general, the model's simulated results show a good convergence based on two standard diagnostics of MCMC simulation, n_eff, and Rhat. The n_eff represents the effective sample size, which is the number of iterations needed for effective independent samples [8]. If the value is greater than 1000, it is a good signal of a strong correlation between the dependent and independent variables. Rhat value – also known as the Gelman shrink factor and the potential scale reduction factor, shows the convergence of the logarithm [17]. If the value is higher than 1.1, the model is not convergent. The Rhat value is computed using the following mathematical formula [18]:

$$\hat{R}=\sqrt{\frac{\hat{V}}{W}}$$

Where $\hat{R}$ represents the Rhat value, $\hat{V}$ is the estimated posterior variance, and *W* is the within-sequence variance.

### Step 3. Model visual diagnostics

One can aesthetically visualize the convergence diagnostics, posterior distribution, and estimated results. The function bvl_plotTrace() can generate the trace plots of the constructed model.

*R> bvl_plotTrace(model3)*

**Fig. 7:**
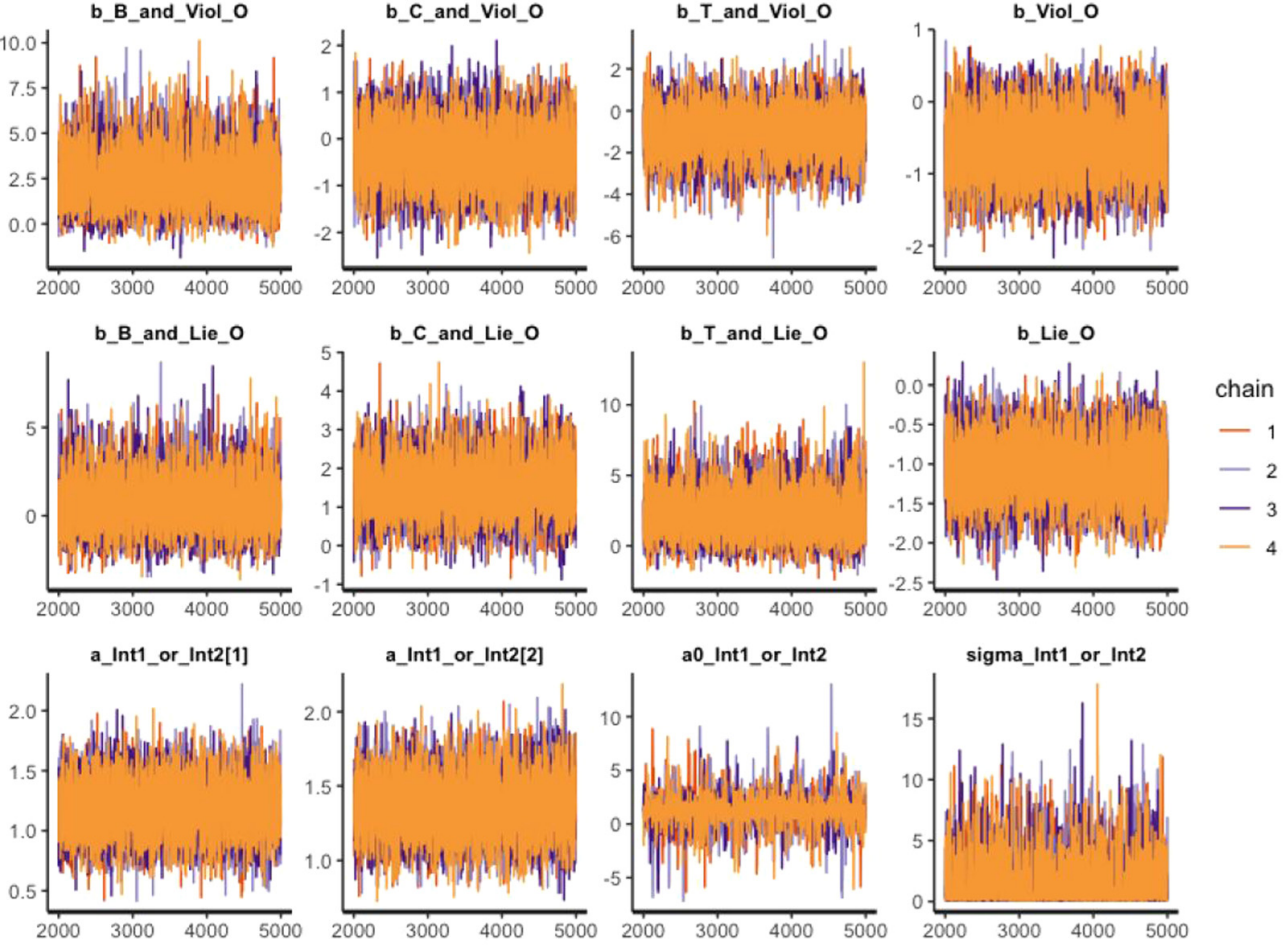
Trace plots of MCMC draws of coefficients in model 3.

Fig. 7 displays the trace plot of each parameter in the model, which is a standard visual diagnostic for MCMC work. The first 2000 samples mark the warmup (adaptation, or burn-in) period. During this period, the Markov chains learn to sample more efficiently from the posterior distribution, so samples in the warmup period are not reliable and representative for inference. It should be noted that the trace plot plotted by the function bvl_plotTrace() only shows the samples after the warmup phase. In order to be identified as "clean, healthy" after the warmup period, the Markov chain needs to meet two primary characteristics: stationarity and good mixing. The chain in Fig. 7 is formed from four component chains, each of which obtains 3000 iterations after the warmup period. Visually, if all lines (or paths) stick around a very stable central tendency, the Markov chain can be considered as stationary, while the rapid zig-zag motions of each line can be seen as the signal for a well-mixing chain. In general, no divergent chains are found, which suggests that the autocorrelation function dies out quickly, and the Markov property is satisfactory with the data distribution at hand. Because the MCMC algorithm produces autocorrelated samples, the function bvl_plotAcfs() is another command to check whether the autocorrelation is eliminated (to 0) after certain finite steps. One can visually diagnose the autocorrelation of the model by the following command, which will generate the results in 4 rows and three columns:

*R> bvl_plotAcfs(model3, NULL, 4, 3)*

**Fig. 8:**
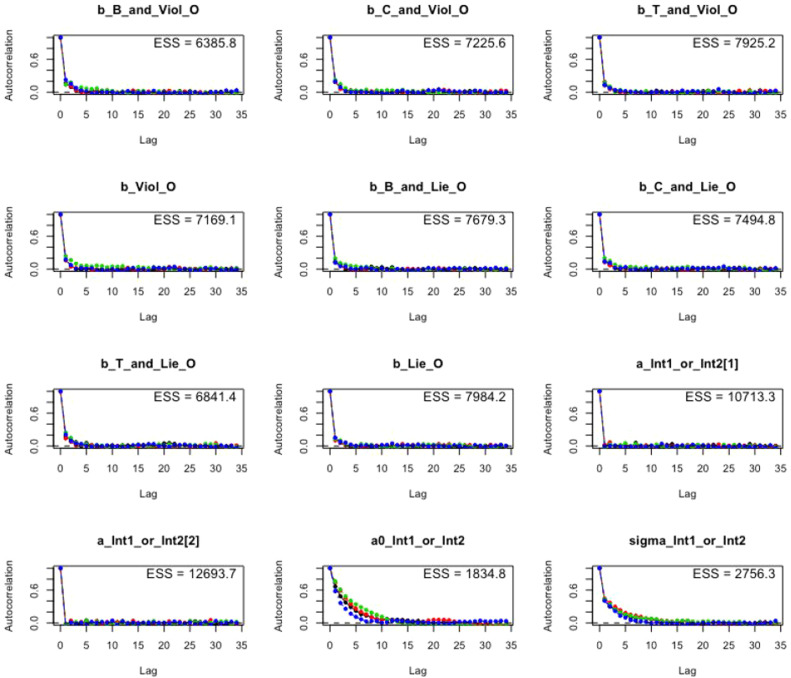
Autocorrelation function plots of coefficients in model 3.

The mathematical formula for the autocorrelation parameter for lag = L is displayed below:

$$ACF_{L}=\left(\frac{T}{T-L}\right)\frac{\sum_{t=1}^{T-L}\left(x_{t}-\bar{x}\right)\left(x_{t+L}-\bar{x}\right)}{\sum_{t=1}^{T}{\left(x_{t}-\bar{x}\right)}^{2}}$$

where *x_t_* is the sampled value of *x* at iteration *t, T* represents the total number of sampled values, and $\bar{x}$ is the mean of sampled values. From Fig. 8, we can see that the effective sample size (ESS), which is all above 1000, reduces quickly to 0 before lag 3. This tendency satisfies the Markov property of the chains and, consequentially, ensure computing efficiency. The Gelman Shrink Factor or the Rhat value estimated above can also be visualized by using the function bvl_plotGelmans():

*R> bvl_plotGelmans (model3)*

**Fig. 9:**
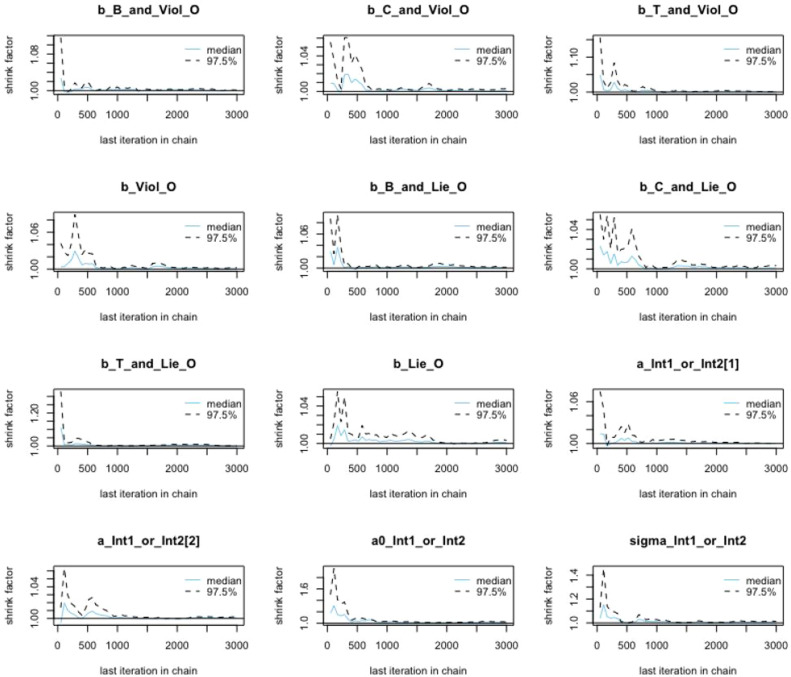
Gelman shrink factor plots of coefficients in model 3.

Measuring how much variance there is between chains relative to how much variance there is within chains is another idea to check the convergence. If the average difference between chains is similar to average difference within chains (when Rhat = 1.0), the chains are well convergent. Nevertheless, the relative value might increase (when Rhat > 1.0) and indicates the less convergent tendency between chains, if there appears at least on orphaned or stuck chain [19]. Fig. 9 illustrates the mean value of potential scale reduction factor for each variable and parameter at 97.5% as well as the shrink factor suggested by Gelman and Rubin [20]. Overall, all the shrink factors get to 1.0 rapidly during the warmup period, which meets the standard of MCMC simulation.

### Step 4. Result of visual presentation

**Fig. 10:**
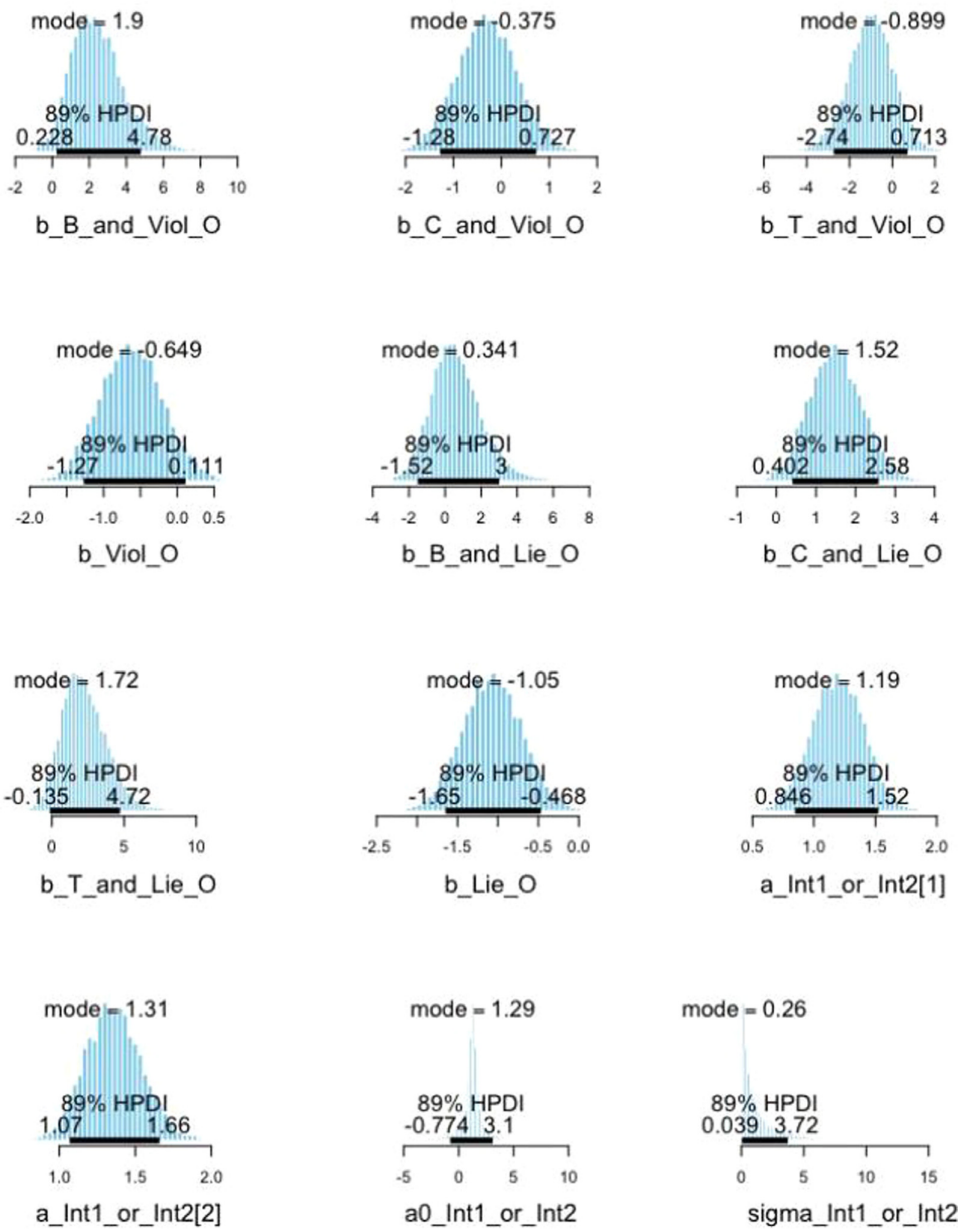
Posterior distribution interval plots of coefficients in model 3.

Besides the mean and standard deviation of the posterior distribution summarized in the model fit above, one can visually present the estimated posterior distribution of every variable coefficient through histograms. The visualization can be made using the function bvl_plotParams(). We visualize the estimated posterior distribution of every variable in the constructed model in four rows and three columns with the Highest Posterior Distribution Intervals (HPDI) at 89% (see Fig. 10). The default HPDI is at 89%; therefore, to adjust the HPDI to 95%, one can simply change the credibility range (credMass) from 0.89 to 0.95.

*R*> bvl_plotParams (model3, row = 4, col = 3, credMass = 0.89, params = NULL)

**Fig. 11:**
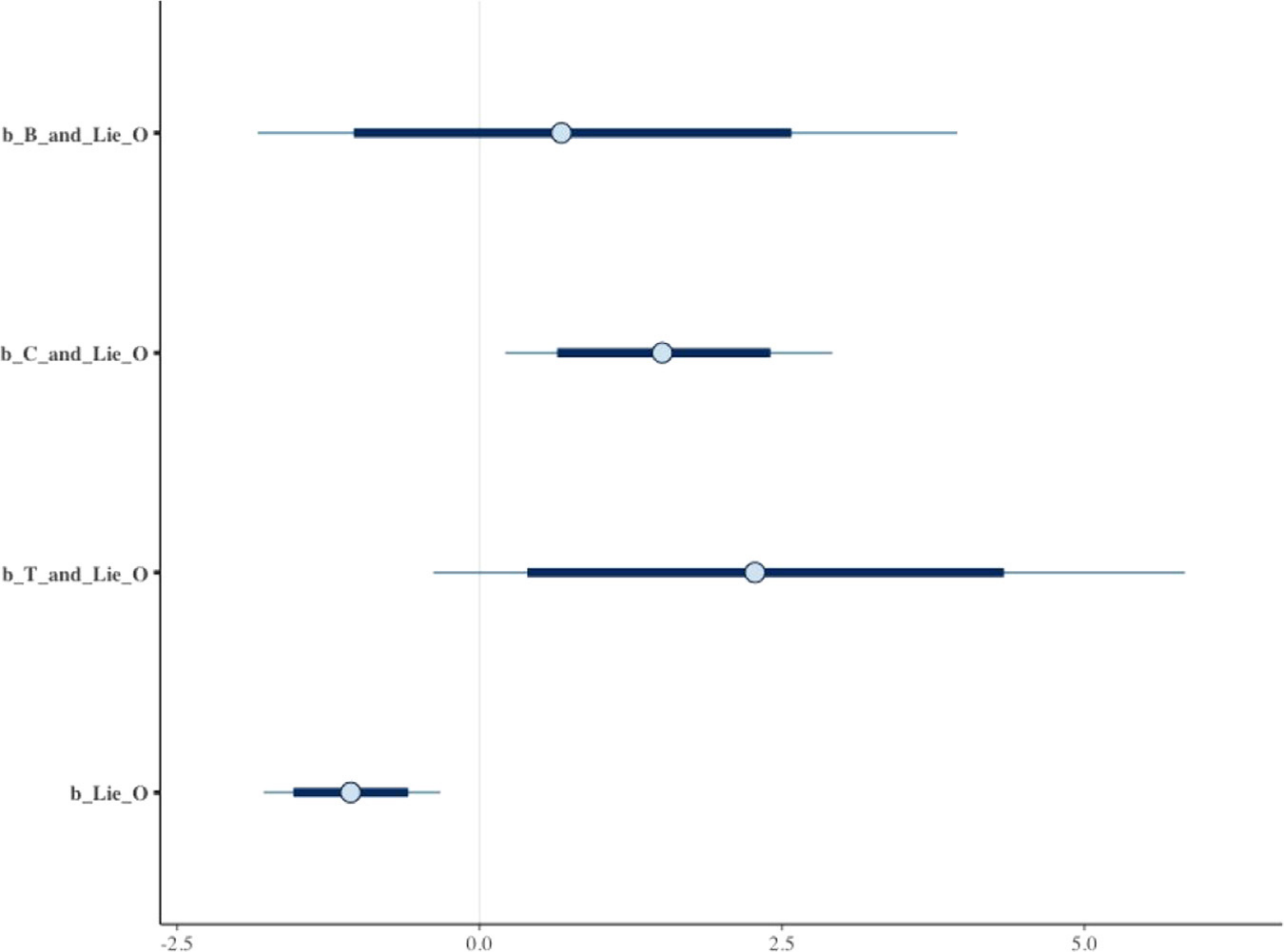
Interval plots of coefficients in model 3.

**Fig. 12:**
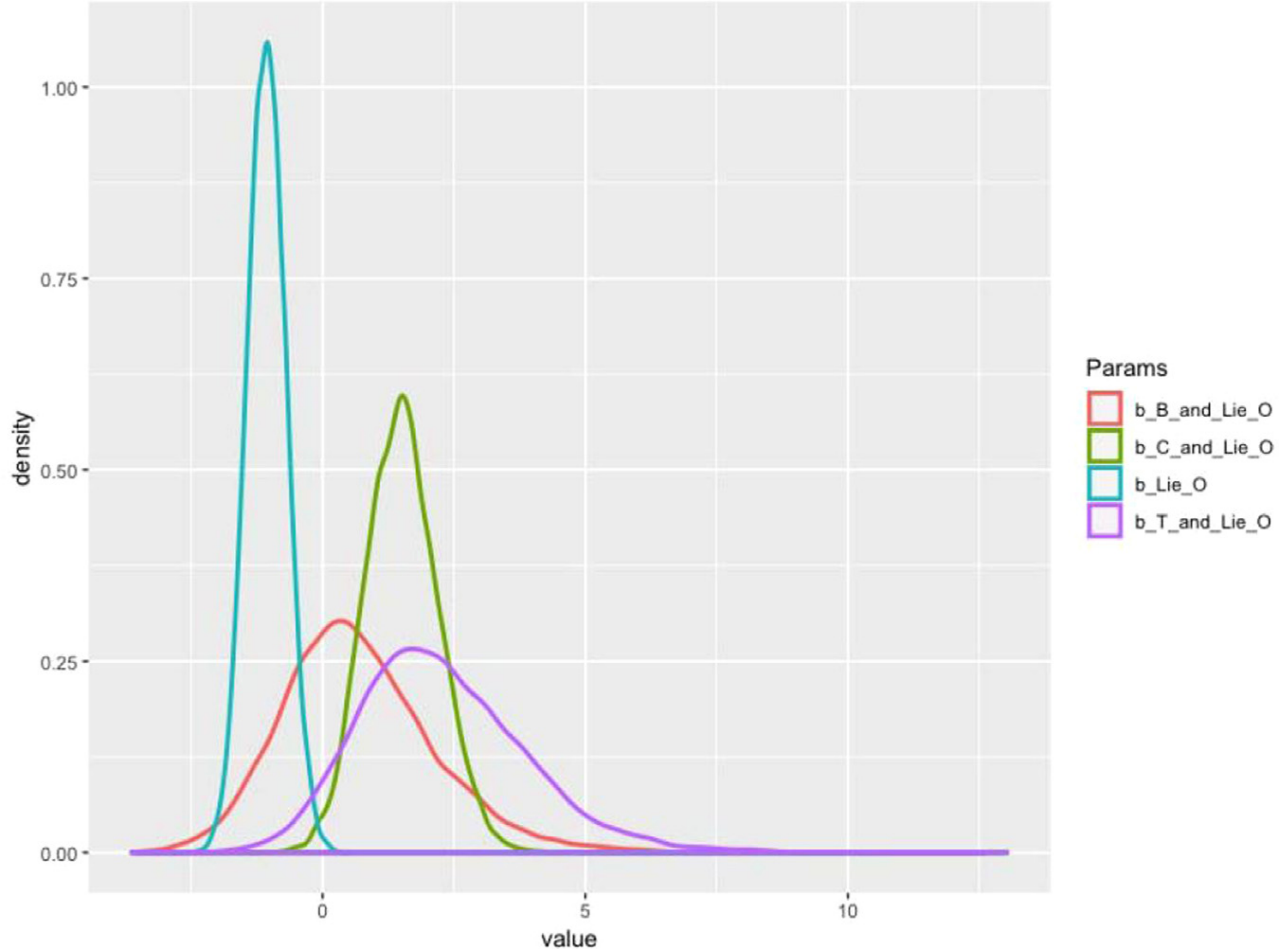
Density plots of coefficients in model 3.

There are also other built-in alternatives to visually present the estimated results after simulation, such as bvl_plotIntervals() and bvl_plotDensity(). The bvl_plotIntervals() function helps visualize the coefficients and their interval, while the bvl_plotDensity() function helps plot the posterior probability density of coefficients. The results can be plotted "all-in-one" or selectively by both functions. The following commands are to visualize the interval (see Fig. 11) and the density (see Fig. 12) of four coefficients ("b_B_and_Lie_O", "b_C_and_Lie_O", "b_T_and_Lie_O", and "b_Lie_O", respectively). If one wants to plot the results by “all-in-one” style, he/she can simply omit c("b_B_and_Lie_O", "b_C_and_Lie_O", "b_T_and_Lie_O", "b_Lie_O").

*R*> bvl_plotIntervals(model3,
+ c("b_B_and_Lie_O", "b_C_and_Lie_O", "b_T_and_Lie_O", "b_Lie_O"))
*R*> bvl_plotDensity(model3,
+ c("b_B_and_Lie_O", "b_C_and_Lie_O", "b_T_and_Lie_O", "b_Lie_O"))

**Fig. 13:**
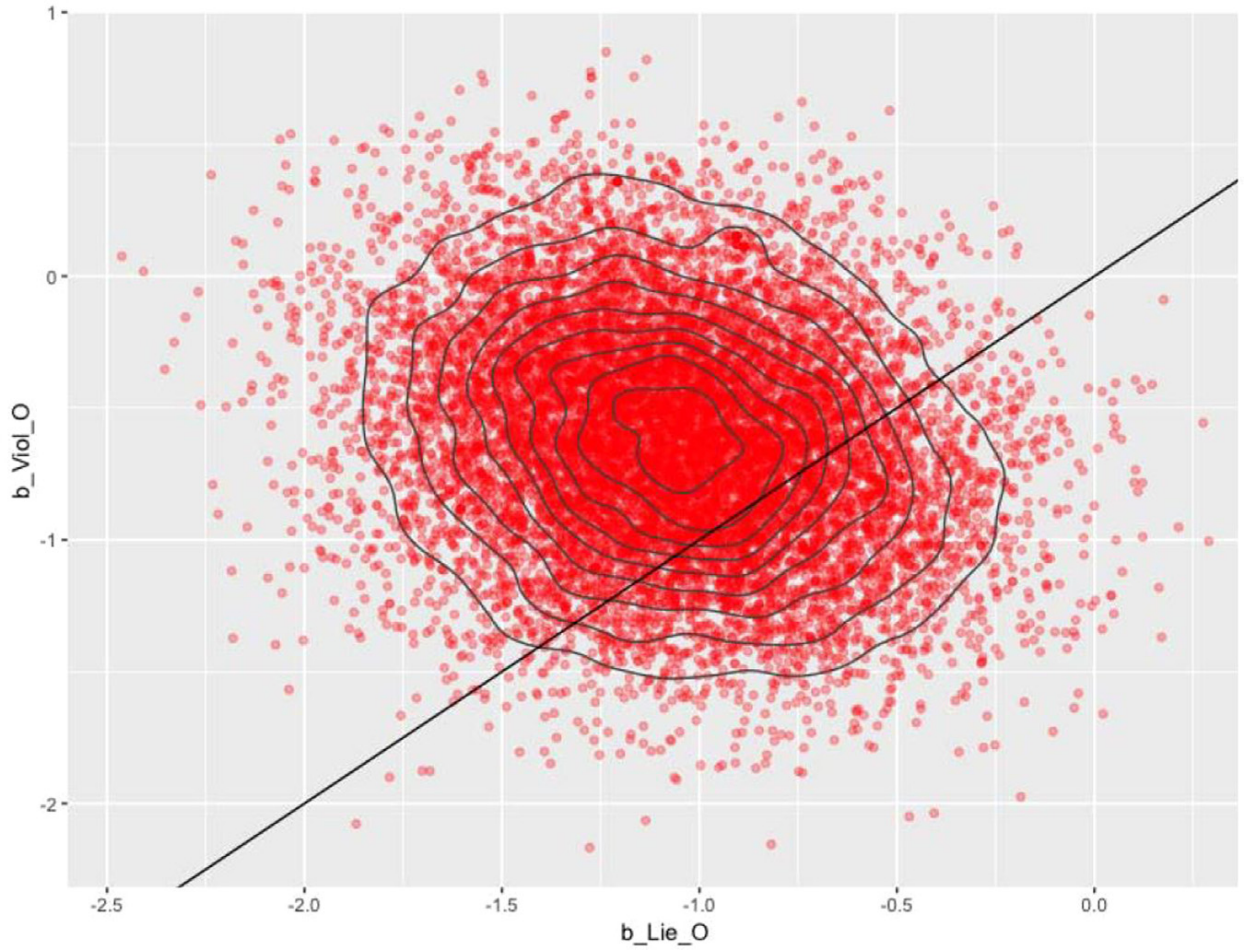
Comparative densities between two "b_Lie_O" and "b_Viol_O".

The comparison between two different coefficients' distribution of posteriors can be plotted by the following code (see Fig. 13):

*R*> bvl_plotDensity2d(model3, "b_Lie_O","b_Viol_O", color_scheme = "red")

## Conclusion

Recently, the reproducibility crisis and the problems of 'stargazing', p-hacking, or HARKing in statistical analysis have required the scientific community to be more rigorous in conducting research and find solutions for the persistent statistical issues. Thus, the method paper proposes Bayesian analysis as a substitution for the conventional frequentist approach. Bayesian statistics have the advantages of treating all unknown quantities probabilistically and incorporating prior knowledge or belief of scientists into the model as an alternative approach for frequentist analysis in social sciences. The usage of the *bayesvl* R package for social data analysis also provides the opportunity to construct a "relationship tree" among variables intuitively and graphically visualize simulated posterior, especially in the age of Big Data [21].

## Declaration of Competing Interest

The authors declare that they have no known competing for financial interests or personal relationships that could have appeared to influence the work reported in this paper.
